# Extracorporeal light-chain elimination in myeloma with simple medium cutoff membrane hemodialysis: a retrospective cohort study

**DOI:** 10.3389/fonc.2023.1193504

**Published:** 2023-09-08

**Authors:** Christian W. Schaaf, Matthias C. Braunisch, Christopher Holzmann-Littig, Frederick Pfister, Liya Hannemann, Renate I. Hausinger, Mareike Verbeek, Christoph Schmaderer, Lutz Renders, Uwe Heemann, Claudius Küchle

**Affiliations:** ^1^ Department of Nephrology, Klinikum rechts der Isar, Technical University of Munich, School of Medicine, Munich, Germany; ^2^ Department of Nephropathology of the Institute of Pathology, School of Medicine, University of Erlangen, Erlangen, Germany; ^3^ Department of Hematology and Oncology, Klinikum rechts der Isar, III. Medical Clinic, School of Medicine, Technical University of Munich, Munich, Germany

**Keywords:** multiple myeloma, dialysis, free light chains, acute kidney injury, medium cutoff hemodialysis

## Abstract

**Background:**

We determined the efficacy of free light chain (FLC) removal by regular dialysis equipment (high-flux filtration) with medium cutoff (MCO) membrane hemodialysis (HD) as an adjuvant treatment to standard chemotherapy for patients with acute kidney injury complicating multiple myeloma (MM) and its impact on further dialysis dependency.

**Methods:**

Sixty patients with acute dialysis-dependent renal failure secondary to MM were treated with MCO-HD (55 patients) or HCO (high cutoff)-HD (5 patients) as a control. FLC serum concentration, total protein, immunoglobulins, and LDH were measured throughout the dialysis therapy. The kidney function of the patients was followed up for 1 year.

**Results:**

The median age was 69 years; 25 female and 35 male patients were enrolled. HD significantly reduced FLC kappa levels in the MCO/HCO group by 58%/84% (MCO/HCO group; *p* < 0.05) and FLC lambda by 39%/33% (MCO/HCO group; *p* < 0.05). Single HD data (MCO) showed a relative reduction of 70% in kappa and 37% in lambda FLC concentration, as expected by the different sizes of the light chains. Renal function improved significantly and continuously from starting creatinine 5.7/3.8 mg/dl (MCO/HCO group) before HD to 1.4/2.0 mg/dl (MCO/HCO group; *p* < 0.001) after 1 year. No significant alteration of total protein, immunoglobulins, and LDH concentrations by HD (HCO and MCO group) was observed. After 1 year, 37 of 60 patients were alive and 34 of them were off dialysis.

**Conclusion:**

FLC elimination with MCO-HD is effective, technically easy, and less cost-intensive as compared with HCO-HD. Kidney function recovery in MM patients is achievable.

## Introduction

In multiple myeloma (MM), therapeutic success strongly depends on the preservation of renal function. Thus, renal impairment represents an independent negative prognostic factor in MM in the first 6 months after diagnosis (1). Accordingly, renal recovery and hematologic response are the strongest markers associated with patient survival (1). This is a major issue since even dialysis-dependent acute kidney injury (AKI) occurs in 10% to 15% of patients with multiple myeloma, and 40% of patients present with a kidney dysfunction at the time of diagnosis (2, 3). In the course of their disease, 50% of patients present with at least one episode of AKI (1, 4).

**Figure 1:**
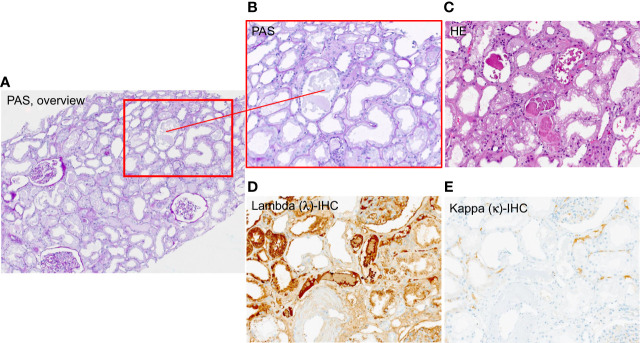
Exemplary histology patient with Multiple Myeloma (MM) showing casts subtype free light chain (FLC) lambda. **(A)** overview 4× magnification PAS-staining, **(B)** PAS-staining 10 × magnification, **(C)** HE-staining 4 × magnification, **(D)** lambda – immunohistochemistry 10× magnification **(E)** kappa – immunohistochemistry 10 × magnification.

A pathophysiological mechanism of damage is the tubulointerstitial cast nephropathy caused by high levels of immunoglobulin free light chains (FLCs) resulting in obstruction, concomitant inflammation, and tubulointerstitial damage (3, 5, 6) (see **Figure 1**). Particularly prone to cast nephropathy are patients with light-chain multiple myeloma due to their high production rate of nephrotoxic FLCs (6, 7). The prognosis of AKI in MM patients used to be very poor, as 80% of affected patients used to remain dialysis-dependent (8).

In animal models, CAST nephropathy causes irreversible damage to the nephron after only 1 month, which indicates a very small, thus, precious timeframe to efficiently reduce FLC in order to allow renal recovery (9, 10).Thus, vigorous efforts have been applied to relieve the impact of myeloma on kidney function, and FLCs seemed to be a reasonable target of therapy. Trials have been conducted since 2005 to remove FLCs by plasmapheresis or later by high cutoff (HCO) membranes, but randomized controlled studies disappointingly did not demonstrate a significant impact on death, dialysis dependency, or reduced kidney function (11, 12). Due to the at-best equivocal results, the high financial expenses, and increased technical requirements for plasmapheresis or high cutoff membrane hemodialysis (HCO-HD), the question arose on how far this approach is mandatory or even applicable.

Nevertheless, in all studies, there seemed to be a group of patients benefiting from the removal of FLCs, as there is a solid pathophysiological rationale for a substantial and particularly rapid FLC removal (1, 2, 13), in order to regain kidney function.

Therefore, we would like to argue for a less lavish, readily available technique to remove FLCs. Here, we report on the results of our focused efforts to eliminate FLCs as quickly as possible through normal dialysis machines with medium cutoff membrane hemodialysis (MCO-HD).

## Materials and methods

### Study design

We performed a retrospective analysis of 75 MM patients treated between August 2015 and June 2021 in the Department of Nephrology, University Hospital rechts der Isar of the Technical University of Munich. In 15 patients, data were not sufficient for assessment. Out of the remaining 60 patients, 55 were treated with MCO membrane (MCO, Theranova 500 PAES/PVP, BPA-free, Baxter, USA), and 5 were treated with HCO membrane (HCO, Gambro THERALITE PAES/PVP, BPA-free, Baxter, USA) dialysis. Assignment was following random selection, and HCO treatment was used as technical control.

Membrane characteristics are the same as described by Boschetti-de-Fierro et al. (14, 15). In order to confirm the effective dialysis of FLCs, we additionally measured concentrations of FLCs in the dialysate on a test basis as proof of principle.

HD was indicated clinically by kappa or lambda light-chain serum levels exceeding 500 mg/L and concurrent acute kidney failure while eliminating alternative diagnosis and proof of tubular cells in urine microscopy. Acute kidney failure was defined as AKI stage 2 (serum creatinine 2.0–2.9 times baseline) or higher following the International Kidney Disease Classification (KDIGO). HD was begun immediately on clinical diagnosis, not waiting for kidney biopsy results.

Most patients were initially treated with an identical chemotherapy regimen (bortezomib-based chemotherapy) according to recent guidelines and the recommendations of the International Myeloma Working Group (16). Depending on the success of the therapy, the chemotherapy regimen was adapted (e.g., cyclophosphamide, daratumumab) following international guidelines at the time of treatment.

The Ethics Committee of the University Hospital rechts der Isar of the Technical University of Munich considered an (extended) ethics vote as not necessary for this study since data were collected for routine purposes; a waiver statement by the Ethics Committee was obtained (number 2/22 S-KH). The study complies with local data protection regulations.

### Data collection

Laboratory data collection was performed using LAURIS laboratory system version 2.21.10 as well as patient records and included basic demographic characteristics (sex, age), disease type, number of dialysis sessions, serum FLC kappa and lambda, serum creatinine, eGFR (CKD-EPI), total protein, immunoglobulin (IgA, IgG, IgM), and LDH concentration at the beginning and end of each session to calculate the relative reduction of these solutes [difference pre–post-dialysis serum concentration divided by pre-dialysis serum concentration * 100% = relative reduction (RR) of these solutes (pre–post)/pre * 100%]. In the case of multiple dialysis sessions, the calculation of relative reduction refers to the difference between the baseline value before starting the first dialysis and the value following the completion of the last dialysis. In the case of the single dialysis data, the calculation of the relative reduction refers to the difference between the baseline value directly before starting this single dialysis and the value following completion of this single dialysis. No patient-identifiable data were collected.

### Statistical methods

Descriptive data of patient characteristics and laboratory values as mentioned above were presented as median and interquartile range (IQR) for continuous variables or frequency of occurrence with proportions for normally distributed and non-parametric data, respectively. Additionally, mean and range were calculated. Depending on the type and the assumed distribution of data, Fisher’s exact test, Mann–Whitney test, or two-way ANOVA were used to analyze the data. For comparison of values before and after dialysis treatment, a two-sample *t*-test for dependent samples (pair comparison test) was performed. A *p*-value ≤0.05 was used as the level of significance, and a *p*-value ≤0.01 was considered highly significant. The data processing was carried out with Microsoft 365^®^ Excel^®^ version 2102 and SPSS version 28.

## Results

### Patient characteristics

Data from 60 patients, treated between August 2015 and June 2021 in the Department of Nephrology, University Hospital rechts der Isar of the Technical University of Munich, were analyzed.

The population was predominantly men (35 patients, 58.3%) and the median age was 69 years (59–78). The primary diagnosis was based on M-protein isotype (2 IgA kappa, 13 IgG kappa, 1 IgM kappa, 3 IgA Lambda, 3 IgD lambda, 10 IgG lambda, 17 kappa light chain only, and 11 lambda light chain only) (**Table 1**). Five patients (8.3%) presented with MM as an initial diagnosis and 55 (91.7%) patients had refractory or relapsing disease (**Table 1**). All patients had high levels of FLC kappa or lambda, and all patients had concomitant AKI. Renal biopsy was performed in 18 cases (30%), confirming CAST nephropathy in 17 cases (see exemplary histopathology of a patient included in this study, **Figure 1**) and AL amyloidosis in one case.

**Table 1:** Overview of patient characteristics.

Patient characteristics	
Number of patients (*n*)	60
Age (years), median (IQR)	69 (59–78)
Age >65 years (%)	30 (50)
Gender (male/female)	35/25
Primary diagnosis (M-protein isotype):	
IgA kappa	2
IgG kappa	13
IgM kappa	1
IgA lambda	3
IgD lambda	3
IgG lambda	10
IgM lambda	0
Kappa light chain only	17
Lambda light chain only	11
Involved sFLC (%):	
Kappa	33 (55)
Lambda	27 (45)
Dialysis with high cutoff membrane number (%)	5 (8.3)
Dialysis with medium cutoff membrane number (%)	55 (91.7)
Histological confirmation of CAST nephropathy number (%)	17 (28.3)
Histological confirmation of AL-amyloidosis number (%)	1 (1.7)
Disease relapse number (%)	55 (91.7)

### Effect of MCO- vs. HCO-HD regarding FLC concentrations

In both MCO and HCO groups, patients received on average eight HD sessions, each session lasting 4 h. For all patients, FLC concentrations were measured repeatedly, at least before and after the HD sessions. For 10 patients, we report on single dialysis data with measured FLC concentrations directly before and after every single HD treatment.

### Effect of MCO- and HCO-HD sessions

**Figure 2:**
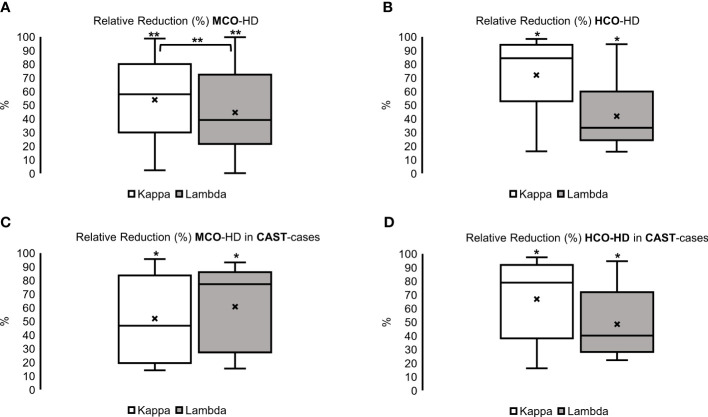
Relative reduction (%) of kappa and lambda FLC over all with **(A)** Medium Cut-Off Hemodialysis (MCO-HD) and **(B)** High Cut-off Hemodialysis (HCO-HD) as well as histologically confirmed CAST cases with **(C)** MCO-HD and **(D)** HCO-HD; Relative Reductions (RR) are calculated as pre-post dialysis serum concentration divided by pre-dialysis serum concentration *100 %; *p < 0.05, **p < 0.01 each.

Overall, MCO- and HCO-HD resulted in a significant relative reduction of FLC concentrations (**Figures 2A, B**, upper part) with no significant difference between MCO- and HCO-HD treatment and no significant difference between histology-confirmed CAST nephropathies and non-biopsied patients (**Figures 2C, D**, lower part).

In the MCO group, an FLC kappa relative reduction of 58% (30–80) and an FLC lambda relative reduction of 39% (22–72) over the whole HD treatment period (**Figure 2**, left; *p < 0.05, **p < 0.01 each) were achieved. The relative reduction of FLC kappa was significantly higher compared with the relative reduction of FLC lambda (**Figure 2A**, left, upper part, **p < 0.01, and **Supplementary Table 1**).

In the HCO group, an FLC kappa relative reduction of 84% (53–94) and an FLC lambda relative reduction of 33% (24–60) over the whole HD treatment period (**Figure 2B**, right, *p* < 0.05 each, and **Supplementary Table 1**) were observed.

**Figure 3:**
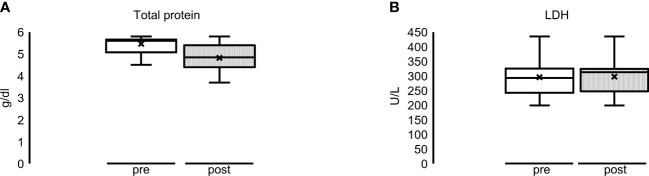
Multiple dialyses with HCO membrane: overall alteration of **(A)** total protein (reduction, not significant) and **(B)** LDH (constant) before (= pre = white bar) and after (= post = striped bar) dialysis sessions.

**Figure 4:**
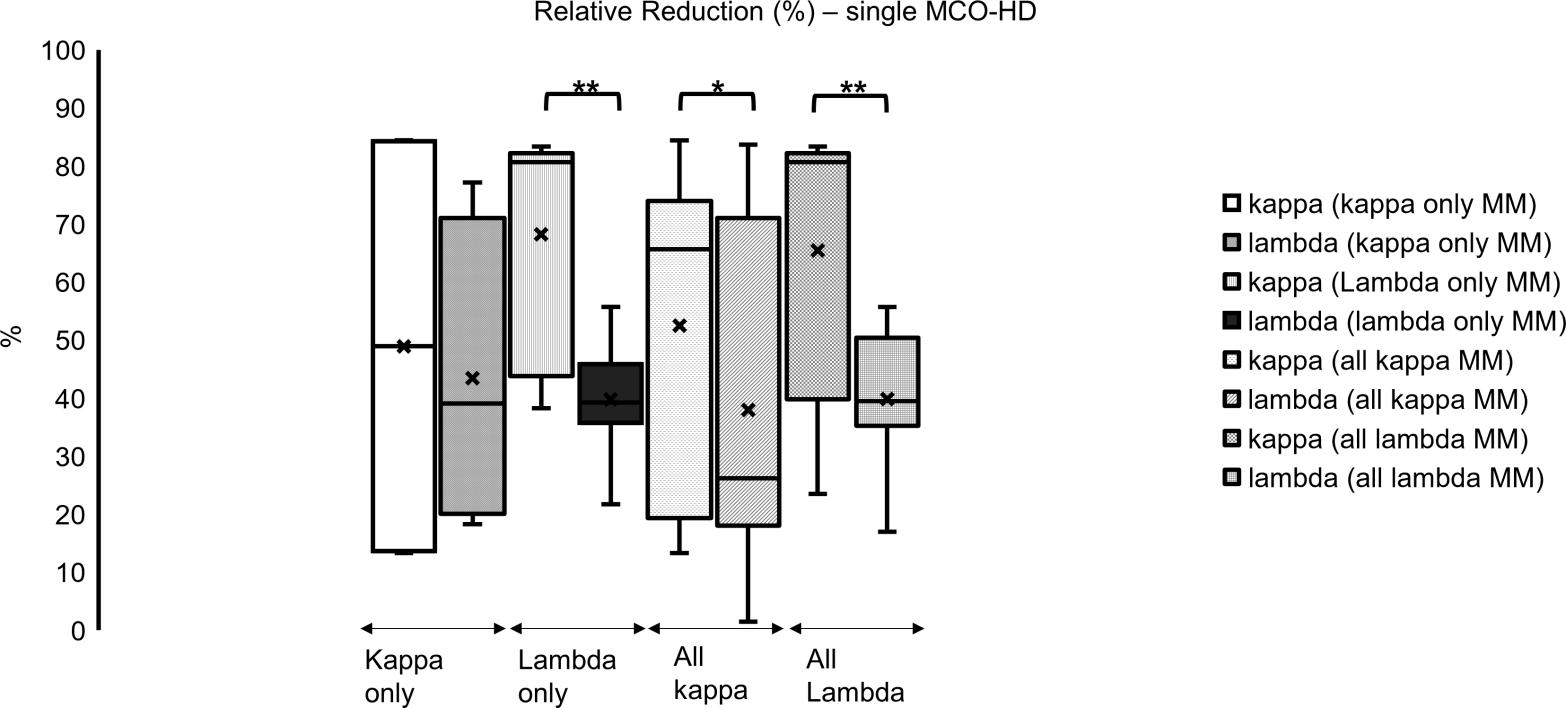
Single dialysis with MCO membrane in different myeloma subtypes: relative reduction of FLC kappa is significantly higher in all subgroups compared to lambda (%); *p < 0.05, **p < 0.001 each kappa vs. lambda FLC reduction.

**Figure 5:**
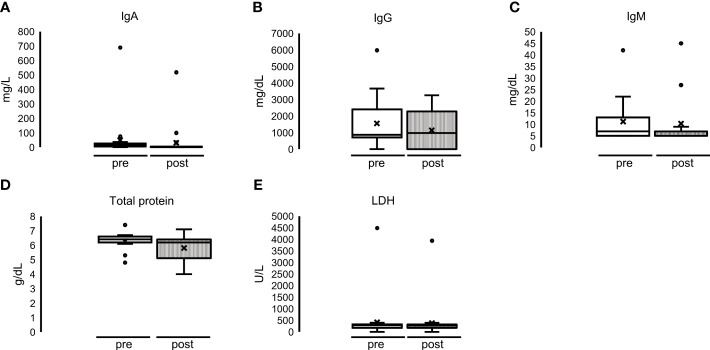
Single dialyses with MCO membrane: no significant alteration of **(A)** IgA, **(B)** IgG, **(C)** IgM, **(D)** total protein and **(E)** LDH before (= pre = white bar) and after (= post = striped bar) MCO-HD.

Regarding the control parameters, the median total protein was reduced in the HCO group from 5.6 g/dl (5.1–5.7) to 4.9 g/dl (4.5–5.4), although not significant. LDH remained approximately the same [293 U/L (243–325) before to 313 U/L (247–325) after the HD treatment period] (**Figure 3**). The same applies to immunoglobulin A, G, and M concentrations (data not shown). Regarding the alterations in the MCO group, we refer to the MCO single dialysis data (see **Supplementary Table 2** and **Figures 4**, **5**).

### Effect of single MCO-HD

**Figure 6:**
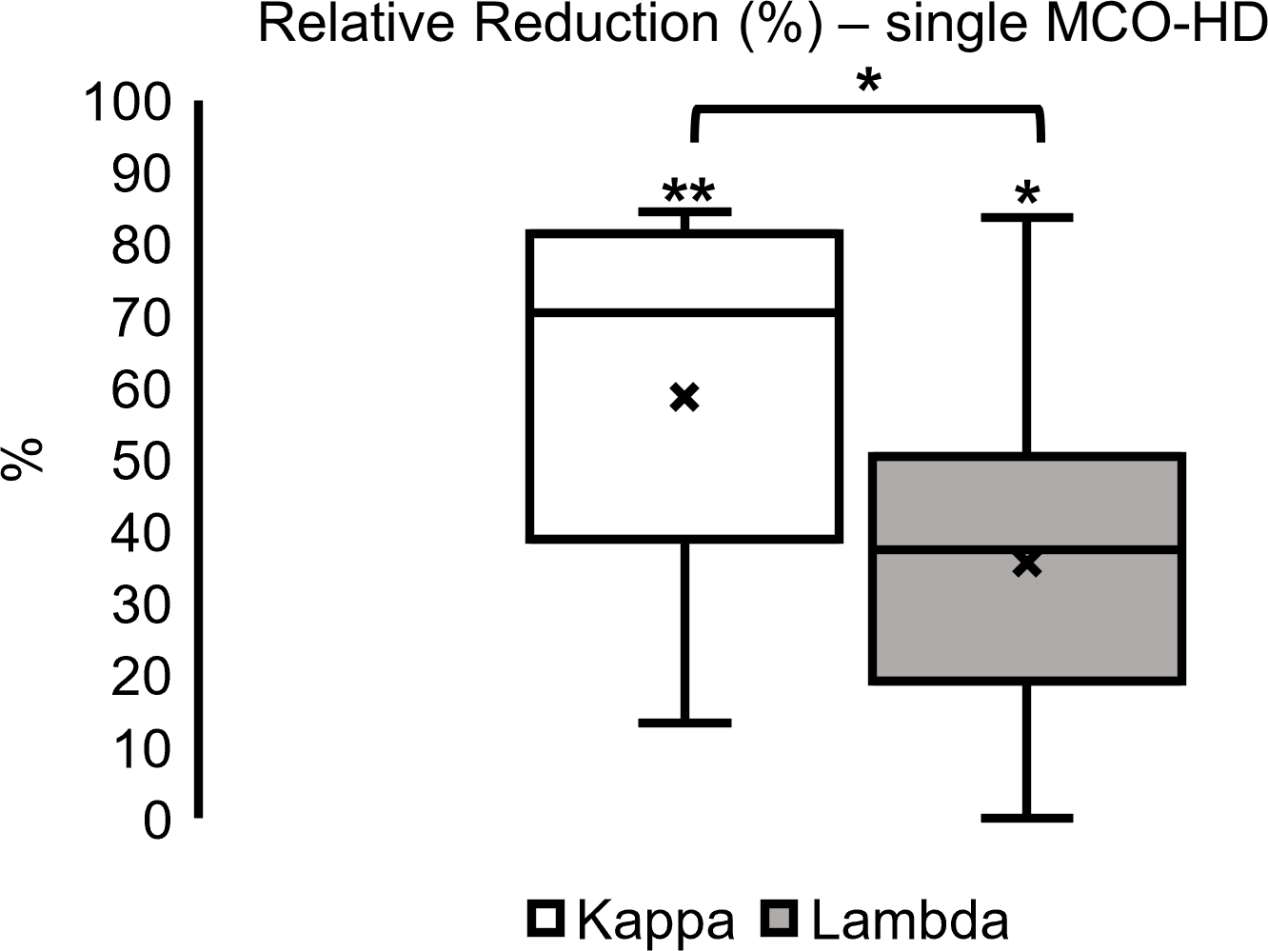
Single dialysis relative reduction (%) in MCO-HD (pre-post)/pre*100%; *p < 0.05, **p < 0.01 each.

Regarding the analysis of single dialysis data, median FLC kappa relative reduction was 70% (39–81) in kappa and 37% (19–50) in lambda FLC concentration over all MM subtypes in these 10 patients. Kappa FLC relative reduction was significantly higher compared with the lambda FLC relative reduction (**Figure 6**; *p < 0.05, **p < 0.01).

### Effect of single MCO-HD on FLC concentrations in different MM subgroups

In the analyzed subgroups of MM (all kappa type MM, only kappa type MM, all lambda type MM, only lambda type MM), the relative reduction (significant) of FLC kappa was higher compared with lambda in all subgroups (mostly significant) (**Figure 4**; *p < 0.05, **p < 0.001 each): all kappa-secreting MM patients, comprising IgG kappa and kappa only cases, showed a median FLC kappa relative reduction of 66% (19–74). In all FLC lambda-secreting MM patients, comprising IgG lambda, IgD lambda, and lambda only cases, an FLC lambda median relative reduction of 39% (35–50) was found.

Looking at the only FLC kappa-secreting myeloma, an FLC kappa relative reduction of 49% (14–84) was identified. Looking at the only FLC lambda-secreting myeloma, an FLC lambda relative reduction of 39% (36–46) was observed (**Figure 4**; *p < 0.05; **p < 0.001 each).

Altogether, the concentrations of FLC kappa and lambda could be reduced with a single MCO-HD significantly, and the relative reduction of FLC kappa was higher compared with FLC lambda.

### Effects on dialysis dependency and kidney function

**Figure 7:**
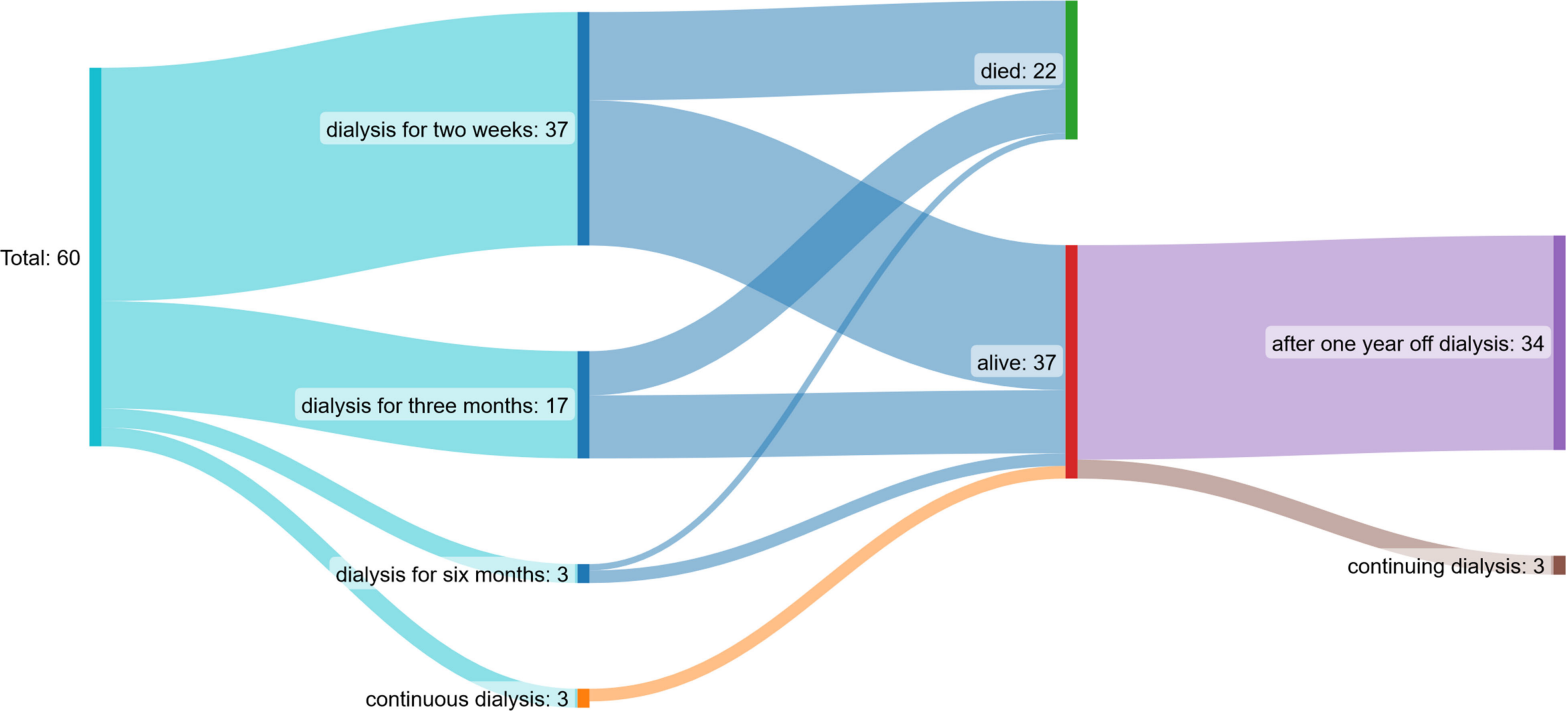
Patient flow chart: 37/60 patients received dialysis sessions for less than two weeks, 17/60 for a period of three months, 3/60 up to 6 months and 3/60 continuously (three times per week) after starting dialysis. After one year, 37 of 60 patients reached follow-up and were alive, 34 were off dialysis.

Renal function 1 year after starting HD improved in both the MCO and HCO groups significantly. Glomerular filtration rate increased and serum creatinine declined, respectively. Thirty-seven of 60 patients received HD sessions for less than 2 weeks, 17/60 for a period of 3 months, 3/60 for up to 6 months, and 3/60 continuously (three times per week) after starting dialysis. In one patient, MCO-HD was stopped because of missing response to the therapy due to the severity of bone marrow infiltration. The patient was continued on regular HF-HD. Thus, after 1 year, 37 of 60 patients reached follow-up and were alive, and 34 were off dialysis. The others died throughout the follow-up period because of myeloma-associated complications (**Figure 7**).

**Figure 8:**
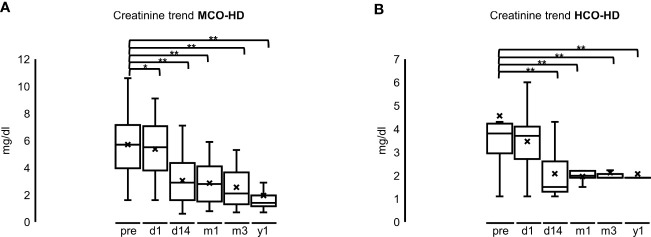
**(A)**: Creatinine trend after MCO-HD; pre = pre MCO-HD, d1 = 1 day, d14 = 14 days, m1 = 1 month, m3 = 3 months, y1 = 1 year after first MCO-HD *p < 0.05; **p < 0.001 each vs. pre-HD. **(B)**: Creatinine trend after HCO-HD; pre = pre MCO-HD, d1 = 1 day, d14 = 14 days, m1 = 1 month, m3 = 3 months, y1 = 1 year after first HCO-HD *p < 0.05; **p < 0.001 each vs. pre-HD.

Renal function recovered earlier (14 days after starting HD) in the HCO group but was similar 1 year after the start of HD compared with the MCO group. Median serum creatinine at the start of HD was 5.7 mg/dl (4.0–7.2) in the MCO and 3.8 mg/dl (3.0–4.2) in the HCO group, 5.5 mg/dl (3.8–7.1) in the MCO and 3.7 mg/dl (2.7–4.1) in the HCO 1 day after starting HD, 3.0 mg/dl (1.6–4.4) in the MCO and 1.5 mg/dl (1.3–2.6) in the HCO 14 days after starting HD, 2.8 mg/dl (1.5–4.1) in the MCO and 2.0 mg/dl (1.9–2.2) in the HCO after 1 month, 2.1 mg/dl (1.3–2.7) in the MCO and 2.1 mg/dl in the HCO after 6 months, and 1.4 mg/dl (1.2–2.0) in the MCO and 2.0 mg/dl in the HCO 1 year after starting HD (**Figures 8A, B**; *p < 0.05; **p < 0.001 each). There were no significant differences between the different MM subgroups.

### Effect of single MCO-HD on serum concentration of immunoglobulins, total protein, and LDH

Although there was a slight decrease in serum concentration of the control parameters total protein and LDH, there were no significant alterations: immunoglobulin concentrations did not differ significantly either: median IgA serum concentration 26 mg/dl (5–26) before HD and 5 mg/dl (5–5) after HD. The same was for IgG: 867 mg/dl (811–2,436) before HD and 977 mg/dl (977–2,294) after HD; IgM (mg/dl) 7 mg/dl (5–13) before HD and 7 mg/dl (5–7) after HD (**Figure 5**, upper part, and **Supplementary Table 2**). The median serum concentration of total protein was 6.5 g/dl (6.2–6.6) before HD and 6.2 g/dl (5.2–6.5) after HD. The median serum concentration of LDH before dialysis was 300 U/L (243–341), and the concentration after HD was 292 U/L (249–320) (**Figure 5**, lower part, and **Supplementary Table 2**).

## Discussion

It is clear that kidney dysfunction adds a great load of morbidity on patients with myeloma, and it is of concern as it is present in 50% of patients during the course of their disease (1–4). Excessive serum levels of FLCs rapidly induce tubulointerstitial damage known as cast nephropathy and thereby pose the risk of dialysis-dependent AKI with a subsequent poor outcome (1, 7, 17, 18). The objective is clear: from the Nordic Myeloma Study Group, we know that the survival of patients with recovered renal function is comparable to patients without AKI (19).

Thus, since 2005, the removal of FLCs by plasmapheresis was attempted but failed to significantly impact death, dialysis dependency, or reduced kidney function (11, 12). Hutchison evaluated the combination of extended high cutoff membrane hemodialysis (HCO-HD; cutoff 45 kDa) and chemotherapy (cyclophosphamide/thalidomide/vincristine/doxorubicin and dexamethasone), which led to a sustained reduction in serum FLC concentrations in the majority of patients, resulting in a substantial rate of dialysis independence (2). However, only those patients who eventually responded to chemotherapy benefitted from HCO-HD (9). It is worth mentioning that supplementation of calcium, magnesium, and albumin was required due to substantial losses during HCO-HD (9).

Similar results were observed in single-center studies showing that effective chemotherapy in combination with extracorporeal FLC removal by HCO-HD was associated with a fast reduction in serum FLC levels and subsequently a higher rate of recovery of kidney function (20–22). Furthermore, prolonged patient survival and renal recovery correlated with the extent of FLC reduction by the HCO membrane (23).

Consequently, the strategy has been further assessed in two multicenter randomized controlled trials: The first trial, MYRE (Multiple Myeloma and Renal Failure due to Myeloma Cast Nephropathy), failed to show a statistically significant effect for the use of HCO-HD in patients treated with bortezomib and dexamethasone, regarding HD independence at 3 months (primary endpoint: 33% vs. 43%, respectively; *p* = 0.42), but an increased renal recovery rate was found later on. As had been previously reported, phosphate and albumin supplementation after HCO-HD was needed (7, 24).

The second trial, EuLITE (European Trial of Free Light Chain Removal by Extended Hemodialysis in Cast Nephropathy), demonstrated that HCO-HD for sessions lasting 6–8 h compared with those receiving high-flux hemodialysis (HF-HD) did not improve clinical outcomes in patients with multiple myeloma and biopsy-proven myeloma cast nephropathy, being on bortezomib, dexamethasone, and doxorubicin treatment (17, 24). Furthermore, there was no additional effect on renal recovery (17, 24). Worse still, the mortality rate after 2 years and the rate of infections were higher in the HCO-HD group. Consequently, a phase 3 study was not pursued (17).

We can assume that these studies failed to demonstrate sound benefits, as the start of therapy took around 10 days after diagnosis (17); thus, CAST nephropathy might already have caused irreversible damage to the kidney. In accordance with this consideration, previous studies found that a rapid reduction of serum FLC levels does improve renal recovery and dialysis independence (7, 18).

There is some sort of consensus that a substantial FLC reduction is mandatory at least in patients at high risk for irreversible renal failure (1, 2, 13, 18). Since the timely response to chemotherapy is somewhat unpredictable, it should be prudent to lower FLC levels by removal as fast as possible. To our knowledge, this is the first study evaluating the clinical experience with a rapid MCO membrane-based hemodialysis.

In our study, MCO-HD significantly reduced serum FLC concentrations and with a similar impact compared with the HCO-HD. Overall, the effect on FLC kappa was stronger than on lambda (58% kappa relative reduction vs. 39% lambda relative reduction over all MCO-HD sessions, highly significant), probably because lambda FLC tended to form covalent dimers, trimolecular complexes, and tetramers with consecutively increased molecular weight, on average 45 kDa for a dimer (25). Consequently, these large molecules exceed the pore radius of MCO membranes with an average cutoff of 40 kDa (14). Furthermore, these multimers even exceed to some extent the pore radius of HCO membranes, which explains the lower relative reduction of FLC lambda compared with kappa in our HCO group (kappa 84% vs. lambda 33%) (15). Thus, it is remarkable that we still achieved a substantial reduction of FLC lambda by MCO-HD.

As in the aforementioned efforts and also in our patients, a significant removal of FLCs and an improvement of renal function after HCO/MCO-HD were found. The renal recovery we found 1 year after starting FLC elimination is in line with previous publications underlining the effectiveness and importance of FLC elimination (4, 26).

Additionally, no significant effects on total protein and immunoglobulin (IgA, IgG, IgM) concentrations could be found in the MCO group, indicating no need for protein or immunoglobulin supplementation or risk of infections due to loss of immunoglobulins. Of note, in the small HCO group, there was an apparent but not significant reduction of total protein, which is in line with protein loss, higher infections, and hemorrhage complications after prolonged HCO-HD in the MYRE study and which therefore supports the preference of alternative MCO-HD (7).

It is worth mentioning that after the first chemotherapy cycle of bortezomib and dexamethasone, only about 30% of patients reached a serum FLC level of less than 500 mg/L, which is considered a critical threshold for cast formation (6, 7). Thus, if there is any doubt about a sufficiently fast response to chemotherapy, starting a technically easy MCO-HD is our proposal based on the observed data. Hereby, the critical timeframe until chemotherapy takes effect can be bridged and used efficiently. This is particularly the case in MM patients with relapsed disease and concomitant AKI, which is an increasingly observed situation in clinical routine and for which the probability of a profound and rapid FLC reduction by chemotherapy is less foreseeable than among patients with newly diagnosed MM (7). Thus, when acute kidney injury requires dialysis treatment, MCO-HD can easily be chosen.

The obvious limitations of this current study relate to its design as a retrospective cohort study with no untreated control group, since a control group without any dialysis treatment was not feasible due to the inclusion of patients with acute kidney failure only. Since EuLITE did not show any benefit in patients with multiple myeloma and biopsy-proven myeloma cast nephropathy (17, 24), a control group with an HF-HD was not even intended.

Some further limitations of our study deserve discussion. First, this a relatively small single-center study, and it included a cohort of patients mainly with an identical chemotherapy regimen (bortezomib- based chemotherapy) according to recent guidelines and the recommendations of the International Myeloma Working Group (16). Our results are in line with the findings of Ramos Terrades et al. who used a large pore size (BK F PMMA, Toray Japan) membrane with the same intention. At the same time, these authors showed that, as expected, HF-HD had no effect on the recovery of kidney function (27). Secondly, a renal biopsy necessary to confirm a CAST nephropathy was not performed in all cases. HD with MCO membrane filtration was indicated clinically by high serum levels of FLCs—kappa or lambda—and concurrent AKI while eliminating alternative diagnosis. Third, in the abovementioned dialysis setting, we could not separate the effect of chemotherapy vs. that of the extracorporeal removal of FLC kappa and lambda.

## Conclusion

Extracorporeal FLC elimination in multiple myeloma patients with straightforward MCO-HD can remove both FLC kappa and lambda subtypes from circulation. In order to improve renal outcome, the time of initiation is critical. In combination with chemotherapy, MCO-HD improves kidney function and reduces renal damage. MCO-HD is as effective as the largely abandoned HCO-HD at a far lesser expense and with fewer side effects. Needless to say, randomized clinical trials and clinical study registers with large cohorts would be necessary to confirm our results. Our data indicate that rapid FLC removal by simple tools is possible and should thus not be denied to MM patients with acute kidney failure.

## Data availability statement

The raw data supporting the conclusions of this article will be made available by the authors, without undue reservation.

## Ethics statement

The studies involving humans were approved by the Ethics Committee of the University Hospital rechts der Isar of the Technical University of Munich. The studies were conducted in accordance with the local legislation and institutional requirements. The Ethics Committee/Institutional Review Board waived the requirement of written informed consent for participation from the participants or the participants’ legal guardians/next of kin because an (extended) ethics vote was not necessary for this study since data were collected for routine purposes; a waiver statement by the Ethics Committee was obtained (number 2/22 S-KH).

## Author contributions

CK contributed to the research idea and study design. CWS, CK are responsible for data collection. CWS, MB and CHL contributed to data analysis and statistics. CWS and CK were mainly involved in writing the manuscript. FP has provided histological images. Each author contributed in critical revision of the article. All authors contributed to the article and approved the submitted version.
