# Supplementary material for: Extracorporeal light-chain elimination in myeloma with simple medium cutoff membrane hemodialysis: a retrospective cohort study
Source: Front Oncol. 2023 Sep 8;13:1193504. doi: 10.3389/fonc.2023.1193504 (PMC10514899; doi:10.3389/fonc.2023.1193504)
Supplement: Supplementary file 1 [file Table_1.docx]

| **HCO group (5 patients)** |  | median | IQR | mean | range |
| --- | --- | --- | --- | --- | --- |
| FLC kappa before dialysis (mg/L) | | 2996 | 1680 - 13113 | 6992 | 15 - 19144 |
| FLC kappa after dialysis (mg/L) | | 300 | 142-1272 | 1450 | 12,4 - 9054 |
| FLC lambda before dialysis (mg/L) | | 12 | 0,95 - 16,5 | 2648 | 0,95 - 23842 |
| FLC lambda after dialysis (mg/L) | | 7 | 0,8 - 11,4 | 505 | 0,7 - 5217 |
|  |  |  |  |  |  |
| **MCO group (55 patients)** |  | median | IQR | mean | range |
| FLC kappa before dialysis (mg/L) | | 90 | 13,5-3715 | 2424 | 5,1 - 40209 |
| FLC kappa after dialysis (mg/L) | | 33 | 6,2-781 | 1550 | 1-27396 |
| FLC lambda before dialysis (mg/L) | | 61 | 10,0-2840 | 4265 | 0,8 - 48492 |
| FLC lambda after dialysis (mg/L) | | 39 | 7,0-911,3 | 1761 | 0,8-20854 |

Supplement

Table 1: FLC serum concentrations of High Cut-Off (HCO)- and Medium Cut-Off (MCO)-HD group before and after dialysis sessions with median, IQR, mean and range
