# Supplementary material for: Extracorporeal light-chain elimination in myeloma with simple medium cutoff membrane hemodialysis: a retrospective cohort study
Source: Front Oncol. 2023 Sep 8;13:1193504. doi: 10.3389/fonc.2023.1193504 (PMC10514899; doi:10.3389/fonc.2023.1193504)
Supplement: Supplementary file 2 [file Table_2.docx]

Supplement

Table 2: Laboratory parameters regarding single dialysis measurements of ten patients treated with Medium-Cut-Off hemodialysis (before and after each single dialysis)

| Laboratory parameters (single dialysis) | | | |  |  | median | IQR | mean | range |
| --- | --- | --- | --- | --- | --- | --- | --- | --- | --- |
|  |  |  |  |  |  |  |  |  |  |
| Free Light Chain (FLC) Kappa (mg/l), before dialysis | | |  |  |  | 25 | 6,1-1758 | 1510 | 4,2-7961 |
| Free Light Chain (FLC) Kappa (mg/l) after dialysis | | |  |  |  | 13 | 1,2-371 | 965 | 0,9-5023 |
| Free Light Chain (FLC) Lambda (mg/l) before dialysis (mg/l) | | | |  |  | 682 | 7,6-1719 | 2544 | 1,3-24554 |
| Free Light Chain (FLC) Lambda (mg/l) after dialysis (mg/l) | | | |  |  | 303 | 6,6-1281 | 1521 | 1,3-16014 |
|  |  |  |  |  |  |  |  |  |  |
| Kappa subtype only (n=4) - FLC Kappa before dialysis (mg/l) | | | | |  | 4660 | 3275-5216 | 3828 | 199-5795 |
| Kappa subtype only (n=4) - FLC Kappa after dialysis (mg/l) | | | | |  | 2230 | 595-4014 | 2379 | 782-5023 |
| all Kappa (n=6) - FLC Kappa before dialysis (mg/l) | | | | |  | 2604 | 336-5216 | 3013 | 32,5 -7961 |
| all Kappa (n=6) - FLC Kappa after dialysis (mg/l) | | | |  |  | 508 | 87-3994 | 1927 | 12,4 - 5023 |
| Lambda subtype only (n=2) - FLC Lambda before dialysis (mg/l) | | | | | | 1457 | 853-2570 | 5313 | 13535-24554 |
| Lambda subtype only (n=2) - FLC Lambda after dialysis (mg/l) | | | | |  | 898 | 529-1561 | 3259 | 7204-16014 |
| all Lambda (n=4)- FLC Lambda before dialysis (mg/l) | | | | |  | 1719 | 1222-3810 | 4866 | 32,5-7961 |
| all Lambda (n=4)- FLC Lambda after dialysis (mg/l) | | | | |  | 1281 | 734-1923 | 2908 | 12,4 - 5023 |
|  |  |  |  |  |  |  |  |  |  |
| Serum creatinine (mg/dl), median (range) - before dialysis | | | | |  | 3,4 | 2,7-5,3 | 4,2 | 1,8-9,1 |
| Serum creatinine (mg/dl), median (range) - 1 day after dialysis | | | | |  | 3,1 | 2,2-3,5 | 3,1 | 1,4-8,5 |
| Serum creatinine (mg/dl), median (range) - 14 days after dialysis | | | | | | 3,1 | 2,2-3,5 | 3,1 | 1,9-4,1 |
| Serum creatinine (mg/dl), median (range) - 1 month after dialysis | | | | | | 2,9 | 2,0-3,3 | 2,7 | 1,3-6,2 |
| Serum creatinine (mg/dl), median (range) - 6 months after dialysis | | | | | | 3,0 | 2,8-3,7 | 2,9 | 1,1-3,7 |
| Serum creatinine (mg/dl), median (range) - 12 months after dialysis | | | | | | 1,7 | 1,7-1,8 | 2 | 1,7-3,1 |
|  |  |  |  |  |  |  |  |  |  |
| Total protein (g/dl) before dialysis | | |  |  |  | 6,5 | 6,2-6,6 | 6,4 | 3,2-7,4 |
| Total protein (g/dl) after dialysis | | |  |  |  | 6,2 | 5,2-6,5 | 5,9 | 4,8-7,1 |
|  |  |  |  |  |  |  |  |  |  |
| IgA (mg/dl) before dialysis | | |  |  |  | 26,0 | 5,0-26 | 56 | 9-689 |
| IgA (mg/dl) after dialysis | |  |  |  |  | 5,0 | 5,0-5,0 | 44,6 | 5-518 |
| IgG (mg/dl) before dialysis | | |  |  |  | 867,0 | 867-2440 | 1778 | 382-5676 |
| IgG (mg/dl) after dialysis | | |  |  |  | 977,0 | 977-2294 | 1599,2 | 345-3254 |
| IgM (mg/dl) before dialysis | | |  |  |  | 7,0 | 5,0-13 | 11,2 | 5-42 |
| IgM (mg/dl) after dialysis | | |  |  |  | 7,0 | 5,0-7,0 | 10,3 | 5-45 |
|  |  |  |  |  |  |  |  |  |  |
| LDH (U/L) before dialysis | | |  |  |  | 299,5 | 243,3-340,8 | 492,6 | 171-4485 |
| LDH (U/L) after dialysis | |  |  |  |  | 291,5 | 248,3-320 | 483,8 | 171-3939 |
